# Ozone-Induced Rice Grain Yield Loss Is Triggered via a Change in Panicle Morphology That Is Controlled by *ABERRANT PANICLE ORGANIZATION 1* Gene

**DOI:** 10.1371/journal.pone.0123308

**Published:** 2015-04-29

**Authors:** Keita Tsukahara, Hiroko Sawada, Yoshihisa Kohno, Takakazu Matsuura, Izumi C. Mori, Tomio Terao, Motohide Ioki, Masanori Tamaoki

**Affiliations:** 1 Center for Environmental Biology and Ecosystem, National Institute for Environmental Studies, Tsukuba, Ibaraki, 305–8506, Japan; 2 Graduate School of Life and Environmental Sciences, University of Tsukuba, Tsukuba, Ibaraki, 305–8577, Japan; 3 Central Research Institute of Electric Power Industry, Abiko, Chiba, 270–1194, Japan; 4 Institute of Plant Science and Resources, Okayama University, Kurashiki, Okayama, 710–0046, Japan; 5 Crop Development Division, Hokuriku Research Center, NARO Agricultural Research Center, National Agriculture and Food Research Organization, Joetsu, Niigata, 943–0193, Japan; China National Rice Research Institute, CHINA

## Abstract

Rice grain yield is predicted to decrease in the future because of an increase in tropospheric ozone concentration. However, the underlying mechanisms are unclear. Here, we investigated the responses to ozone of two rice (*Oryza Sativa* L.) cultivars, Sasanishiki and Habataki. Sasanishiki showed ozone-induced leaf injury, but no grain yield loss. By contrast, Habataki showed grain yield loss with minimal leaf injury. A QTL associated with grain yield loss caused by ozone was identified in Sasanishiki/Habataki chromosome segment substitution lines and included the *ABERRANT PANICLE ORGANIZATION 1 (APO1)* gene. The Habataki allele of the *APO1* locus in a near-isogenic line also resulted in grain yield loss upon ozone exposure, suggesting *APO1* involvement in ozone-induced yield loss. Only a few differences in the APO1 amino acid sequences were detected between the cultivars, but the *APO1* transcript level was oppositely regulated by ozone exposure: i.e., it increased in Sasanishiki and decreased in Habataki. Interestingly, the levels of some phytohormones (jasmonic acid, jasmonoyl-L-isoleucine, and abscisic acid) known to be involved in attenuation of ozone-induced leaf injury tended to decrease in Sasanishiki but to increase in Habataki upon ozone exposure. These data indicate that ozone-induced grain yield loss in Habataki is caused by a reduction in the *APO1* transcript level through an increase in the levels of phytohormones that reduce leaf damage.

## Introduction

Tropospheric ozone is the main photochemical oxidant that causes extensive damage to cultivated crops [1]. Its concentration has increased markedly since the turn of the past century [2]. It is predicted that the concentration will continue to increase in Eastern Asia until 2020, where it may trigger up to 40% crop yield loss [3]. Acute exposure to ozone results in foliar lesions such as chlorosis and necrosis and induces a variety of biochemical and physiological responses in plants [4–6]. Ozone enters the leaves through the stomata, resulting in the generation of reactive oxygen species (ROS) through an oxidative burst [7]. The ROS induce programmed cell death with a result that resembles the hypersensitive response provoked by pathogen infection [4].

Yield loss in ozone-exposed crops is thought to be triggered by a reduction in photosynthetic activity and by growth inhibition caused by leaf injury [8, 9]. The latter has been assessed in this context in many rice cultivars [10–12], and the mechanism of leaf damage induction has been clarified: for instance, two QTLs associated with leaf bronzing identified using two rice cultivars Nipponbare (ozone-sensitive *japonica* cultivar) and Kasalath (ozone-tolerant *indica* cultivar) differed significantly in leaf ascorbic acid content when exposed to ozone, suggesting ascorbic acid as a principal antioxidant counteracting ozone-induced oxidative damage [11]. However, the degree of visible ozone-induced leaf injury does not correlate with grain yield reduction in 20 rice cultivars [13], suggesting that ozone-induced grain yield loss in rice may not be accounted for by the reduction in photosynthetic activity caused by leaf damage. Several genes involved in rice grain yield have been identified by quantitative trait locus (QTL) analysis [14–17]. For example, the transcript level of the cytokinin oxidase/dehydrogenase gene (*Gn1a/OsCKX2*) in inflorescence meristems regulates the number of reproductive organs and grain yield [14]. *GW2*, encoding a RING-type E3 ubiquitin ligase, controls rice grain width and weight; GW2 negatively regulates cell division by targeting its substrate(s) for proteasomal degradation [16]. Yet despite the identification of several such genes, genes involved in ozone-induced grain yield loss in rice have not been identified, although the QTL associated with relative dry weight was found using two cultivars Nipponbare and Kasalath, which this QTL exhibited a less reduced net photosynthetic rate under ozone exposure compared with Nipponbare [11].

The objectives of this study were to identify QTLs associated with rice grain yield loss under elevated ozone by using Sasanishiki/Habataki chromosome segment substitution lines (CSSLs). One year QTL analysis showed that a QTL associated with rice grain yield loss by ozone-exposure was located on chromosome 6 [18]. Here, we carried out further experiments and show that the *ABERRANT PANICLE ORGANIZATION 1* (*APO1*) gene, known to control panicle branching in rice, has important role for ozone-induced grain yield loss. Ozone induces suppression of *APO1* expression during panicle formation, resulting in a reduction in the number of panicle branches and eventually in grain yield. We also carried out further and to clarify how ozone stress-induced signaling regulates grain yield by affecting early morphogenesis.

## Materials and Methods

### Plant materials and growth conditions

To detect QTLs associated with ozone-induced grain yield loss, we used a mapping population consisting of 39 CSSLs developed from rice (*Oryza sativa* L.) cultivars Sasanishiki (*japonica* cultivar, recurrent parent) and Habataki (*indica* cultivar, donor parent) [19]. Seeds were sown in plastic boxes (28 cm × 21 cm × 9 cm; 80 plants per box) filled with seedbed soil, and the seedlings were grown in a glasshouse under ambient air at the Akagi Testing Center of the Central Research Institute of the Electric Power Industry (Gunma Prefecture, Japan, 36°28′ N, 139°11′ E, 540 m above sea level). Six weeks after sowing, the seedlings were transplanted into pots (0.05 m^2^ surface area and 0.015 m^3^ volume; four plants per pot) and grown in glasshouses (five pots of each line per glasshouse) under ambient air or elevated ozone. For the latter treatment, artificially generated ozone was added to ambient air via a mass flow controller. Plants were grown until harvest from 10 April to 25 September 2009 and from 28 April to 28 September 2010. The mean ozone concentrations during the daytime (6:00 to 18:00) were 32.0 nL L^−1^ in 2009 and 43.7 nL L^−1^ in 2010 in ambient air, and 76.5 nL L^−1^ in 2009 and 85.7 nL L^−1^ in 2010 in ozone-supplemented air (Fig A in S1 File). Average of air temperature, relative humidity and light intensity (photosynthetically active radiation) in both glasshouses were not significantly difference throughout growing period in 2009 and 2010 (data not shown). For total RNA isolation, plants were pulled from the pots at 23 and 10 days before heading, and leaves, roots, young panicles (~5 cm long) and inflorescence meristems (~1 cm long) enclosed by the leaf sheath were frozen at −80°C. Further research on Habataki-genotype *APO1* gene were carried out using the progenies of 04SHA422-12-8.8–18.31 [20]. Of these, SHA422-1.1 contains Habataki-genotype of *APO1* gene, and SHA422-1.3 has Sasanishiki-genotype of *APO1* gene. The SHA422-1.1, SHA422-1.3, Sasanishiki and Habataki were grown in an open-top chamber (five pots of each line per chamber) under charcoal-filtered air or elevated ozone from 13 May until harvest on 30 September 2011. The mean ozone concentration during the daytime (6:00 to 18:00) was 6.0 nL L^−1^ in charcoal-filtered and 67.0 nL L^−1^ in ozone-supplemented air (data not shown). Mean temperature and relative humidity in the open-top chamber were 22.8–24.2°C and 80–84.7%, respectively. The obtained results in 2011 were converted to equivalent of NF (non-filtered air) condition using conversion factors calculated from growth traits of Sasanishiki, Habataki, and SL421 grown at open-top chamber in 2010 (Table A in S1 File).

### QTL analysis

Yield and plant growth parameters were measured as described previously [18] and those in parental lines are listed in Table B in S1 File. Linkage analysis was performed by interval mapping [21] as implemented in the program R/qtl [22], using the expectation-maximization algorithm [23]. The genotype of each CSSL was determined previously [19]; the mapping data were obtained from the Rice Genome Resource Center (http://www.rgrc.dna.affrc.go.jp/). Recombination fractions were converted to centimorgans (cM) by using the Haldane mapping function [24]. Putative QTLs were also detected using R/qtl.

### Sequence analysis of *APO1* gene

Genomic DNAs extracted from Sasanishiki and Habataki seedlings by using a DNeasy Plant Mini Kit (Qiagen, Valencia, CA, USA) were amplified by PCR using the *APO1*-specific primers APO1-F2 (5’–ATGATGAACCCTCGCCGGCTGC–3’) and APO1-full-R (5’–CTAACCATCATGCATGCCATGCAAGGCG–3’). PCR products were purified by using a QIAquick Gel Extraction Kit (Qiagen) and cloned into the pDrive Cloning Vector (Qiagen PCR Cloning Kit; Qiagen). Cloned amplicons were sequenced on an ABI3730xl DNA analyzer (Life Technologies, Carlsbad, CA, USA). These experiments were carried out following the manufacturer’s instructions. Functional motifs (F-box domain and Kelch motif) were predicted by SWISS-MODEL [25–27].

### Quantitative PCR analysis

Total RNAs were extracted from frozen samples (leaves, roots, young panicles and inflorescence meristems) by using RNeasy Plant Mini Kit (Qiagen). First-strand cDNA was generated from total RNA using random hexamer primers (Invitrogen, Carlsbad, CA, USA) and used as a template for quantitative PCR with the *APO1*-specific primers 51L3 (5’–CAGGTAAGGGCTCCGTTGGA–3’) and 53R3 (5’–TGCGTAGCATGTTTTGCAGT–3’) [20]. A fragment of the α-tubulin gene was also amplified from the same cDNA with the primers TUB-F (5’–CATCGACATCAAGTTCGA–3’) and TUB-R (5’–CCGAGTTCGACGATGGTGA–3’), and used as an internal standard to estimate the relative expression level of *APO1* gene. These experiments were carried out following the manufacturer’s instructions.

### Microarray analysis

Total RNAs were extracted from frozen inflorescence meristems of Sasanishiki and Habataki grown in ambient air condition and elevated ozone condition by using RNeasy Plant Mini Kit (Qiagen). Two hundred ng of total RNAs were subjected to the analysis. A rice 4 x 44K custom oligo-DNA microarray chip (Agilent Technologies, CA) was used for genome-wide gene profiling. Microarray analysis including cDNA synthesis, fluorescence labeling, hybridization, scanning and digitization of gene expression level were performed by a service provider, Hokkaido System Science (http://www.hssnet.co.jp/index_e.htm). The four gene subsets were extracted from microarray data by using these filtering criteria as below: (1) the fold change both in Sasanishiki and Habataki was ≥ 2, (2) that both in Sasanishiki and Habataki were ≤ 0.5, (3) that in Sasanishiki was ≥ 2 and that in Habataki was ≤ 0.5, (4) that in Sasanishiki was ≤ 0.5 and that in Habataki was ≥ 2, compared with ambient air condition. To identify the gene ontology of extracted genes, the RAP Os IDs in subsets were converted to UniProt accession numbers by using DAVID 6.7 [28, 29], and the gene enrichment analyses for up- or down-regulated genes were performed by using FuncAssociate 2.0 [30]. The microarray data have been deposited in NCBI's Gene Expression Omnibus (GEO, http://www.ncbi.nlm.nih.gov/geo/) and are accessible through GEO Series accession number GSE65465.

### Quantification of phytohormone contents

The content of phytohormones (indole-3-acetic acid, IAA; *trans*-zeatin, tZ; N6-isopentenyladenine, iP; abscisic acid, ABA, gibberellins A_1_, GA_1_; gibberellins A_4_, GA_4_; jasmonic acid, JA; jasmonoyl-l-isoleucine, JA-Ile; and salicylic acid, SA) was determined according to the method of Lehisa and co-workers [31] with modifications. Frozen inflorescence meristems and flag leaves (~200 mg) were ground to a fine powder, mixed with 4 mL of 80% (v/v) acetonitrile containing 1% (v/v) acetic acid and known amounts of stable isotope-labeled internal standards, and stored for 1 h at 4°C to extract the hormones. Tissue debris was pelleted by centrifugation at 3000 ×*g* for 10 min, and the pellet was washed with 80% (v/v) acetonitrile containing 1% (v/v) acetic acid. The two supernatants were combined, evaporated in a vacuum centrifugal evaporator (Sakuma, EC-57CS, Tokyo, Japan) and dissolved in 1% (v/v) acetic acid. The extracted hormones were loaded onto a reverse-phase solid-phase extraction cartridge (Oasis HLB 1 cc; Waters Corporation, Milford, MA, USA). The cartridge was washed with 1 mL of 1% acetic acid and hormones were eluted with 2 mL of 80% acetonitrile containing 1% acetic acid. The eluent was evaporated to leave the extracts in 1 mL of 1% acetic acid and subjected to cation exchange chromatography on an Oasis MCX 1-cc extraction cartridge (Waters Corporation). The cartridge was successively washed with 1% acetic acid and 80% acetonitrile. The acidic fraction was eluted with 1 mL of 80% acetonitrile containing 1% acetic acid. A portion of the acidic elute was analyzed for SA as detailed below. The cartridge was further washed with 5% aqueous ammonia, and the basic fraction was eluted with 40% acetonitrile containing 5% ammonia and analyzed for tZ and iP. The remaining acidic fraction was evaporated, dissolved in 1% acetic acid, and loaded onto an Oasis WAX 1-cc extraction cartridge (Waters Corporation Inc.). The cartridge was washed with 1% acetic acid and the remaining hormones were eluted with 80% acetonitrile containing 1% acetic acid. The elute was analyzed for IAA, GA_1_, GA_4_, ABA, JA, and JA-Ile.

All fractions were analyzed on an Agilent 1260–6410 Triple Quad LC/MS system (Agilent Technologies Inc., Santa Clara, CA, USA) equipped with a ZORBAX Eclipse XDB-C18 column (Agilent Technologies Inc.). The conditions of liquid chromatography are described in Table C in S1 File. The multiple-reaction-monitoring mode of the tandem quadrupole mass spectrometer and precursor-product ion transitions for each compound are listed in Table D in S1 File.

### Statistical analysis

All statistical analyses were conducted by use of the open source software R version 3.1.1 [32, 33].

## Results

### Differences in grain yield and plant growth parameters in Sasanishiki and Habataki under elevated ozone and QTL analysis

**Fig 1:**
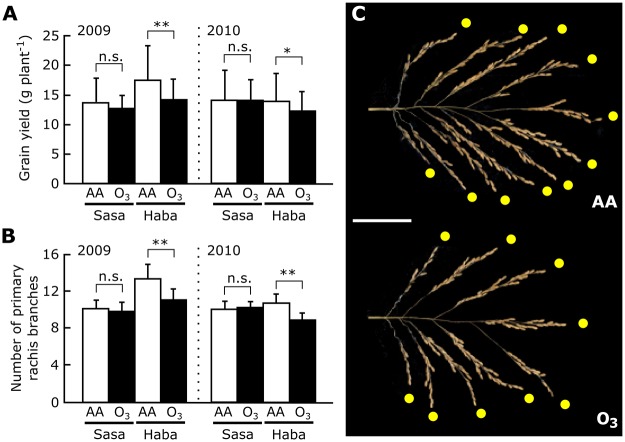
Effects of elevated ozone on two rice cultivars, Sasanishiki and Habataki. (A) Changes in grain yield in 2009 and 2010. (B) Changes in the number of primary rachis branches in 2009 and 2010. Values are mean ± SD (*n* = 20). Error bars indicate SD; n.s., not significant; *P<0.1; **P<0.05 (Student’s *t*-test). AA, ambient air; O_3_, elevated ozone; Sasa, Sasanishiki; Haba, Habataki. (C) Typical panicles of Habataki grown under ambient air (left) or elevated ozone (right). Yellow circles indicateidentify each primary rachis branches. Scale bar = 5 cm.

We first investigated changes in vegetative and reproductive traits caused by ozone exposure in Sasanishiki and Habataki. In Habataki, ozone exposure (approximately twice the concentration in the ambient air) reduced grain yield by 19% (*P* = 0.038) in 2009 and by 12% (*P* = 0.085) in 2010 relative to control plants (Fig 1A), although no or weak leaf injury was detected (Table B in S1 File) [18]. By contrast, no ozone-induced reduction of grain yield was observed in Sasanishiki. Interestingly, visible leaf injury appeared in ozone-exposed Sasanishiki (Table B in S1 File) [18]. The number of primary rachis branches was significantly decreased (by 17%) upon ozone exposure in Habataki but not in Sasanishiki (Fig 1B and 1C). Ozone-induced changes in other vegetative or reproductive traits (biomass, culm length, panicle number per plant, panicle length, sterile grain number, total grain number, number of filled grains per panicle, and filling rate) were observed in both cultivars (Table B in S1 File). However, ozone-induced changes in these traits were detected only in one of the two years in both cultivars. Therefore, we consider only the number of primary rachis branches and grain yield as traits affected by ozone in Habataki but not in Sasanishiki, and these traits were assessed further.

**Fig 2:**
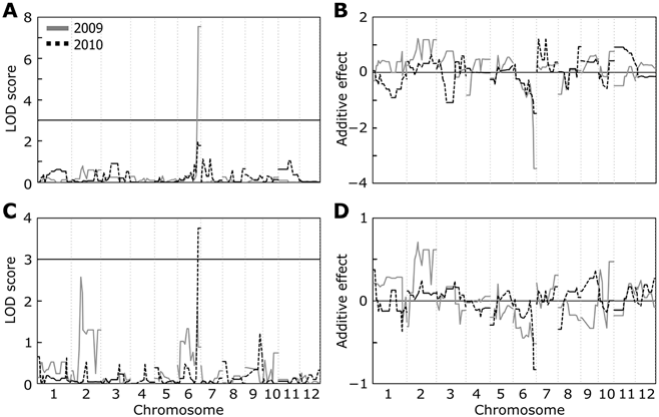
Genome scans for ozone-induced yield loss and the number of primary rachis branches. (A, C) QTL likelihood maps for (A) grain yield and (C) the number of primary rachis branches. Genetic maps were produced by composite interval mapping using differences between ambient air and elevated ozone. (B, D) Additive effect of (B) QTLs for grain yield and (D) the number of primary rachis branches. A positive (negative) additive effect in *B* and *D* represents an increasing allele from Sasanishiki (Habataki). The vertical dotted lines separate chromosomes 1–12 (labeled at the bottom) progressing left to right along the *x*-axis.

**Fig 3:**
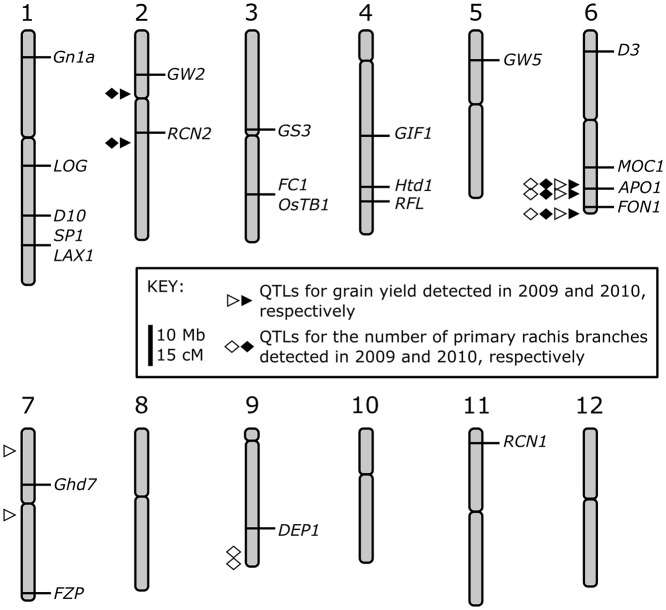
A genetic linkage map showing the positions of QTLs for grain yield and for the number of primary rachis branches on rice chromosomes in 2009 and 2010. The map is adapted from [43]. Genes known to affect grain yield in rice are indicated on the right of each chromosome.

We performed QTL analyses for these traits in the CSSLs and found QTLs on chromosome 6 involved in ozone-induced reduction of grain yield and of the number of primary rachis branches (Fig 2A and 2C). The LOD score for grain yield was > 7 in 2009 (Fig 2A); although it was <3 in 2010, this value was still the largest among all chromosomes. The LOD score of the QTLs for the number of primary rachis branches was highest in 2010 (> 3), although it was not the highest (< 2) in 2009 (Fig 2C). The highest peak was detected at the end of short arm in chromosome 2 (Figs 2 and 3), but the additive effect of this QTL indicates an increasing allele from Sasanishiki (Fig 2). The positions of the QTLs for grain yield and the number of primary rachis branches at the end of chromosome 6 were nearly identical (Fig 2). The negative additive effect of these QTLs indicates an increasing allele from Habataki (Fig 2B and 2D).

### Effects of Habataki-genotype of *APO1* on grain yield and number of primary rachis branches

**Fig 4:**
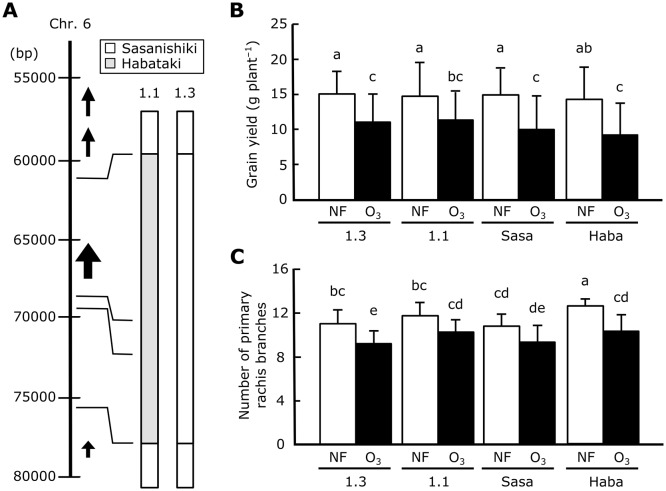
Effects of the Habataki-type *APO1* gene in Habataki and the SHA422-1.1 near-isogenic line. (A) Graphical genotype of chromosome 6 of SHA422-1.1 (*APO1* near-isogenic line) and SHA422-1.3. The thickest arrow represents the open reading frame of *APO1*; narrower arrows represent other predicted genes. 1.1, SHA422-1.1; 1.3, SHA422-1.3. Modified from [20]. (B, C) Effects of the Habataki-type *APO1* gene on (B) grain yield and (C) the number of primary rachis branches. Values are mean ± SD (*n* = 36). NF, non-filtered air (converted values); O_3_, elevated ozone. Bars topped by the same letters are not significantly different (Tukey’s HSD test, *P*<0.05).

The highest LOD scores for grain yield and the number of primary rachis branches in 2009 and 2010 were at the RM3430 marker (107.6 cM on chromosome 6; Figs 2 and 3). These results suggest that genes near RM3430 play key roles in grain yield loss by decreasing the number of primary rachis branches under elevated ozone. Our previous study showed that RM3430 lies close to the *ABERRANT PANICLE ORGANIZATION 1* (*APO1*) gene [18]. The gene encodes an F-box protein [34] and is known to affect rice grain yield through regulation of primary rachis branch formation [20]. To investigate whether the *APO1* allele in Habataki is involved in the decrease in grain yield and in the number of primary rachis branches, we carried out an ozone exposure experiment with SHA422-1.1, a near isogenic line derived from a CSSL that has only the Habataki-genotype *APO1* region in the Sasanishiki background (Fig 4A). As a control, we also used SHA422-1.3, a sib line of SHA422-1.1 that almost all region of chromosome have the Sasanishiki genotype. Grain yield and plant growth parameters of SHA422-1.1, SHA422-1.3, Sasanishiki and Habataki grown in the open-top chambers with elevated or low (charcoal-filtered air) ozone were measured (Fig 4B, 4C and Table E in S1 File). Grain yield and the number of primary rachis branches decreased significantly under elevated ozone in all of three lines, except grain yield of SHA422-1.1 (*P* = 0.059, Fig 4B and 4C). Both grain yield and the number of primary rachis branches decreased significantly under elevated ozone in SHA422-1.1 and Habataki (*P*<0.05, Fig 4B and 4C). These results indicate that the Habataki allele of *APO1* is involved in the reduction of grain yield and of the number of primary rachis branches by ozone.

### Accumulation of *APO1* transcripts in Habataki is suppressed by ozone

**Fig 5:**
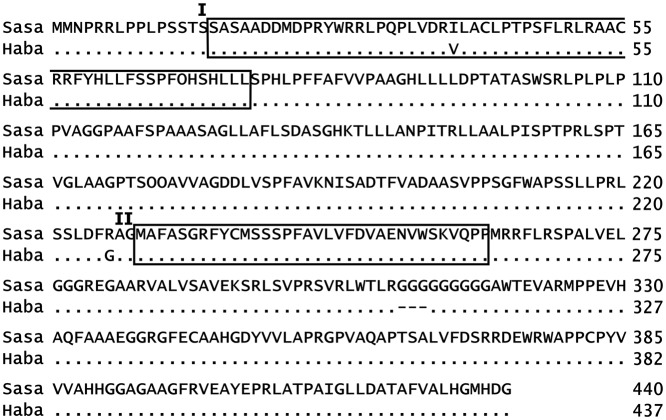
APO1 amino acid sequences in Sasanishiki and Habataki. Boxes show predicted functional motifs (I, F-box domain; II, Kelch motif). APO1 of Habataki has two amino acid substitutions (Ile39Val in the F-box domain and Arg226Gly near the Kelch motif), and a deletion of three amino acids (Gly309–Gly311) in comparison with Sasanishiki. Sasa, Sasanishiki; Haba, Habataki.

We speculated that the primary structure of the APO1 protein, the expression level of its gene, or both differ between Sasanishiki and Habataki. First, we sequenced the *APO1* alleles in Sasanishiki and Habataki and found two amino acid substitutions (I39V and R226G), and a deletion of three sequential glycine residues (from 309 to 311) in Habataki (Fig 5). APO1 has an F-box domain and a Kelch motif, which are involved in its enzymatic activity [35]. The substitution I39V in the F-box domain might affect protein function. To examine this possibility, we performed 3D homology modeling of the F-box domain and found that this substitution would not interfere with the 3D structure of the F-box domain (Fig B in S1 File), indicating that there is no functional difference between the APO1 F-box domains in Sasanishiki and Habataki.

**Fig 6:**
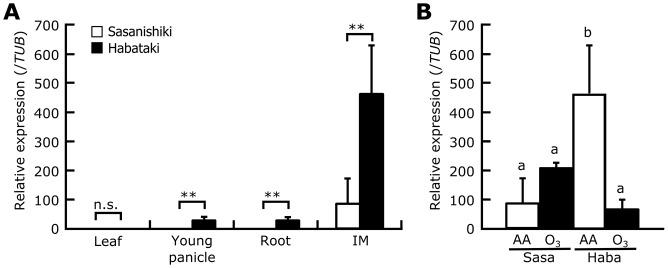
Relative levels of *APO1* transcript in different organs. (A) *APO1* transcript levels in the fourth leaf, young panicle (10 days before heading), root, and an inflorescence meristem (IM; 23 days before heading). (B) Ozone-induced changes in the *APO1* transcript level in inflorescence meristems of Sasanishiki (Sasa) and Habataki (Haba). Values are mean ± SD (*n* = 3). AA, ambient air; O_3_, elevated ozone. n.s., not significant; **P<0.05 (Student’s *t*-test, A). Bars topped by the same letters are not significantly different (Tukey’s HSD test, *P*<0.05, B).

We have previously reported that the level of *APO1* transcripts in young panicles was suppressed by ozone in Habataki but increased in Sasanishiki [18]. To understand the *APO1* expression pattern in more detail, we compared it in several organs in the two cultivars. The *APO1* transcript was detected in the young panicles, roots, and inflorescence meristems, but not in the leaf blades; the expression level was higher in Habataki than in Sasanishiki (Fig 6A). In Habataki, remarkably high *APO1* expression was observed in inflorescence meristems, where it was 17 times that in young panicles. Ozone treatment reduced the *APO1* transcript level in inflorescence meristems of Habataki to one-seventh of that under ambient air, but increased the transcript level in Sasanishiki by approximately 100%, although this increase did not reach statistical significance (Fig 6B, *P* = 0.076). These findings are in line with our previous report for young panicles [18]. Furthermore, the *APO1* transcript level in SHA422-1.1 was 5-fold higher than that in SHA422-1.3 under NF condition, but that in both lines were decreased by ozone treatment (Fig C in S1 File).

### Jasmonic acid and abscisic acid levels are increased in Habataki by ozone exposure

To understand the difference of *APO1* regulation in Sasanishiki and Habataki, we analyzed whole genome gene expression profile in inflorescence meristems of the two cultivars using microarray analysis. The four gene subsets were obtained from microarray data (e.g. subset 1; genes up-regulated in both cultivars, subset 2; genes down-regulated in both cultivars, subset 3; genes up-regulated in Sasanishiki but down-regulated in Habataki, subset 4; genes down-regulated in Sasanishiki but up-regulated in Habataki). Twenty-six, 650, 480, and 275 genes were identified by above criteria from subset 1, 2, 3 and 4, respectively, and these were classified into 137, 617, 582 and 279 attributions by their gene ontology (GO). Among of them, 12, 5 and 19 GOs were selected over- or under-represented attributes by gene enrichment analyses from subset 2, 3 and 4, respectively. No GO was identified in subset 1 by this criterion. Genes responsive to oxidative stress were mainly detected in subset 2 (data not shown). The obtained result is reasonable because we carried out microarray analysis in ozone-exposed plants. On the other hand, genes involved in phytohormone response were significantly enriched in subset 4 (Table F in S1 File, *P*<0.05). It is noteworthy that ozone response of genes categorized into subset 4 was opposite to that of *APO1* in inflorescence meristem (Fig 6B). Moreover, previous studies showed that phytohormones, such as cytokinins, JA, and ABA) influence the grain yield of rice [14, 36, 37]. Production of some phytohormones (JA, ABA, ethylene, and SA) in leaves increases upon ozone exposure [38]. These prompted us to investigate the possible involvement of phytohormones in the regulation of *APO1* expression under elevated ozone in rice.

**Fig 7:**
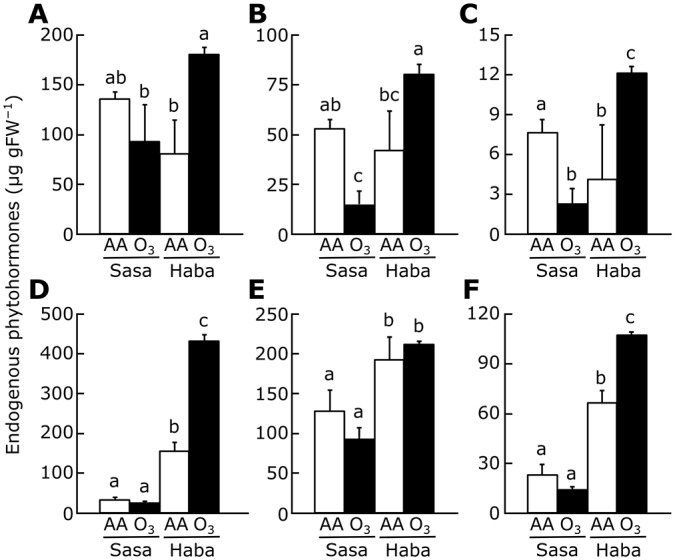
Ozone-induced changes in endogenous phytohormone levels in Sasanishiki and Habataki. The levels of (A, D) ABA, (B, E) JA, and (C, F) JA-Ile were measured in (A–C) inflorescence meristems and (D–F) flag leaves. Values are mean ± SD (*n* = 3). AA, ambient air, O_3_, elevated ozone; Sasa, Sasanishiki; Haba, Habataki. Bars topped by the same letters are not significantly different (Tukey’s HSD test, *P*<0.05).

We quantified the levels of phytohormones (IAA, tZ, iP, ABA, GA_1_, GA_4_, JA, JA-Ile, and SA) in inflorescence meristems and flag leaves with or without exposure to elevated ozone. We could not detect GA_1_ or GA_4_ in either cultivar. Although the levels of IAA, tZ, iP, and SA differed between Sasanishiki and Habataki, the contents of these phytohormones in inflorescence meristems and flag leaves were not affected by ozone in either cultivar (except for iP and SA in flag leaves, but the significant difference of these phytohormones were observed only one of the two cultivars) (Table G in S1 File). Upon exposure to elevated ozone, the levels of ABA, JA, and JA-Ile in inflorescence meristems of Sasanishiki decreased by 31.0% (not significant), 72.9%, and 71.1%, respectively, but increased to 2–3 times the levels under ambient air in Habataki (Fig 7A–7C). The same tendency was also observed in flag leaves: the content of JA, JA-Ile, and ABA in flag leaves was 2–10 times that in inflorescence meristems (Fig 7D–7F). It is noteworthy that the levels of most phytohormones (except IAA and tZ in both cultivars, and ABA and iP in Sasanishiki) were higher in flag leaves than in inflorescence meristems (Fig 7 and Table G in S1 File).

## Discussion

Ozone exposure reduced grain yield (by 12%–19%) and the number of primary rachis branches (by 17%) in Habataki, but not in Sasanishiki. However, the incidence of ozone-induced leaf lesions was higher in Sasanishiki than in Habataki (Table B in S1 File). Our previous reports also showed that grain yield loss in Habataki caused by ozone exposure was not associated with leaf lesions [13, 18]. These data indicate that Habataki is a suitable rice cultivar to clarify the relationship between leaf injury and grain yield loss caused by ozone exposure.

In the QTL analysis for grain yield using plants growing under ambient air or elevated ozone, we found a peak with the maximum LOD score on chromosome 6 in both 2009 and 2010 (Figs 2 and 3). The QTL had a positive effect on grain yield under ambient air in Habataki; this positive effect was not detected under elevated ozone [18]. Thus, the QTL on chromosome 6 appears to be involved in ozone-induced grain yield loss. While narrowing down the position of the QTL for grain yield loss caused by ozone, we found a locus near RM3430 on chromosome 6 which may be involved in the decrease in the number of primary rachis branches under elevated ozone. RM3430 was located close to *APO1*, which is known to affect rice grain yield through regulation of primary rachis branch formation [20]. Indeed, SHA422-1.1, in which *APO1* is the only gene substituted with a Habataki allele in the Sasanishiki background [20], showed a reduction in grain yield and in the number of primary rachis branches under elevated ozone (Fig 4B and 4C). In addition, contribution of *APO1* locus in decrease of grain yield and number of primary rachis branches under elevated ozone were calculated as 57.8% and 66.7%, respectively (data not shown). These data indicate that ozone-induced reduction in grain yield and in primary rachis branch number is mainly controlled by the *APO1* locus of Habataki, although entire of ozone-induced grain yield loss is not explained with a locus.

APO1 amino acid sequences and homology modeling of the functional domain (F-box domain; Fig B in S1 File) suggested that no differences for primary structure of the protein were between Sasanishiki and Habataki. The *APO1* transcript level in inflorescence meristems was suppressed by elevated ozone in Habataki but not in Sasanishiki. Such reduction was also observed in young panicles of Habataki [18]. Taken together, these data suggest that in Habataki, ozone-induced reduction of *APO1* expression in inflorescence meristems (rather than the differences in the APO1 primary structures) might reduce the number of primary rachis branches, resulting in low grain yield. Ozone-induced reduction of *APO1* expression (Fig C in S1 File), grain yield and number of primary rachis branches in SHA422.1.1 (Fig 4B and 4C), which has Habataki-genotype of *APO1* locus, may support the suggestion. The absence of such reduction in *APO1* expression in Sasanishiki is consistent with the absence of any effect of ozone on grain yield or on the number of primary rachis branches in this cultivar.

The F-box domain is often found in phytohormone receptors such as TIR1, COI1, SLY1, and EBF1/2 (receptors for auxin, JA, GA, and ethylene, respectively) [39]. The levels of ABA, JA, and JA-Ile were increased by ozone in inflorescence meristems of Habataki, but were shown tendency to decrease in those of Sasanishiki (Fig 7A–7C). Both JA and ABA, which are produced under drought stress, prevent spikelet formation in rice [37, 40]. However, in this study we found that ozone-induced grain yield loss in Habataki occurred as a consequence of a decrease in the number of primary rachis branches. Treatment with methyl jasmonate decreases grain yield and the number of primary rachis branches in rice [36]. It is likely that the increase in JA or JA-Ile in ozone-exposed Habataki may suppress *APO1* expression in inflorescence meristems, resulting in grain yield loss in this cultivar. Although the role of JA in regulating rice spikelet development has been investigated [41], further studies are needed to understand the mechanism of *APO1* regulation by the JA pathway under elevated ozone.

**Fig 8:**
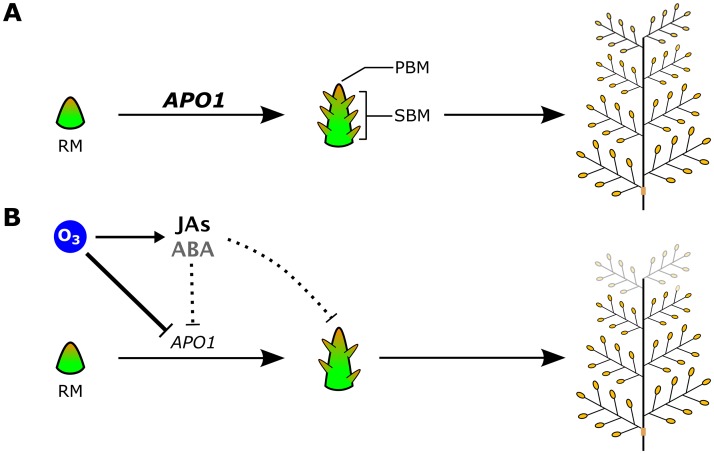
A hypothetical model of ozone-induced grain yield loss in Habataki. (A) Panicle formation flow under ambient air condition. The *APO1* transcription is the trigger of phase transition from rachis meristems (RM) to primary and secondary branch meristems (PBM and SBM). The transcript level is higher in Habataki than Sasanishiki, this is the reason Habataki is high-yielding cultivar. (B) Ozone enters leaves through stomata and generates ROS, which triggers generation of JAs and ABA in leaves to attenuate leaf damage. ROS signaling suppress the *APO1* transcript level directly or indirectly in Habataki. In parallel, phytohormones generated in leaves might be translocated to inflorescence meristems through the phloem. These impacts suppress differentiation of the RM into the PBM and SBM. Consequently, the decrease in primary rachis branch formation reduces grain yield.

JAs generated upon ozone exposure in leaves attenuate ozone-induced leaf injury [36]. This is consistent with our findings that ozone-induced leaf damage was suppressed in Habataki, which had high levels of leaf JAs (Fig 7) [18]. Furthermore, the content of JA, JA-Ile, and ABA in flag leaves was 2–10 times that in inflorescence meristems (Fig 7). These data indicate that JAs generated in Habataki leaves reduce ozone-induced leaf injury, and may also suppress *APO1* expression in inflorescence meristems. By contrast, the effect of *APO1* suppression alone is not completely explains ozone-induced grain yield loss in Habataki. According to our result shown here, two hypothetical pathways of grain yield loss in ozone-exposed rice are considered; (1) ozone suppresses *APO1* expression directly or through JAs and ABA signaling, and (2) JAs (and ABA) signaling suppress phase transition of rachis meristem to primary and secondary branch meristems that is independently occurred to *APO1* suppression. As described, contribution of *APO1* in ozone-induced reduction of grain yield in Habataki was estimated as 58%, suggesting JAs and ABA have the potential of substantial portion of residual 42% impacts for ozone-induced grain yield reduction. Taken together, our findings suggest the following model of ozone-induced grain yield loss in Habataki (Fig 8). Upon plant exposure to elevated ozone during the panicle formation stage, JAs and ABA are generated in leaves to attenuate leaf injury: unfortunately the ABA-mediated mechanism remain unclearly, ABA may (indirectly) attenuate ROS production [42]. Ozone signal(s) suppress *APO1* expression directly, or these phytohormones translocate to inflorescence meristems and suppress *APO1* expression presumably, resulting in a reduction in the number of primary rachis branches and eventually in grain yield.

## Conclusions

Our study indicates that different responses for grain yield in elevated-ozone condition between the two rice cultivars were triggered through a change in panicle morphology controlled by *APO1* gene. Intriguingly, visible injury in ozone-exposed leaves seems to relate to this mechanism consequently via several phytohormones. Our data suggest a unified framework to explain the relationship between leaf damage and reduction of grain yield caused by ozone exposure.

## Supporting Information

S1 File Supporting Tables and Figures. Table A in S1 File. Grain yield and number of primary rachis branches (PRB) grown at OTC in 2010, and conversion factors of CF to NF conditions. Table B in S1 File. Effects of exposure to elevated ozone on grain yield and plant growth parameters in Sasanishiki and Habataki. Table C in S1 File. LC conditions. Table D in S1 File. Parameters for LC-ESI-MS/MS analysis (Agilent 1260–6410). Table E in S1 File. Effects of exposure to elevated ozone on grain yield and plant growth parameters in SHA422-1.3, SHA422-1.1, Sasanishiki and Habataki (2011). Table F in S1 File. Overrepresented attributes for the 275 genes that responded under elevated ozone condition found by gene enrichment analysis. Table G in S1 File. Effects of exposure to elevated ozone on the amounts of phytohormones (μg gFW^−1^) in inflorescence meristems and flag leaves. Fig A. in S1 File Daily ozone exposure in the glasshouse in 2009 and 2010. Fig B. in S1 File Backbone ribbon representation of the F-box domains of APO1 from Sasanishiki and Habataki. Fig C. in S1 File Ozone-induced changes in the *APO1* transcript level in inflorescence meristems of SHA422-1.3 and SHA422-1.1.
(PDF)
