# Supplementary material for: Ozone-Induced Rice Grain Yield Loss Is Triggered via a Change in Panicle Morphology That Is Controlled by ABERRANT PANICLE ORGANIZATION 1 Gene
Source: PLoS One. 2015 Apr 29;10(4):e0123308. doi: 10.1371/journal.pone.0123308 (PMC4414449; doi:10.1371/journal.pone.0123308)

Ozone-induced rice grain yield loss is triggered via a change in panicle morphology that is controlled by *ABERRANT PANICLE ORGANIZATION 1* gene

Keita Tsukahara, Hiroko Sawada, Yoshihisa Kohno, Takakazu Matsuura, Izumi C. Mori, Tomio Terao, Motohide Ioki and Masanori Tamaoki.

S1 Supporting Information

S1 Table A. Grain yield and number of primary rachis branches (PRB) grown at OTC in 2010, and conversion factors of CF to NF conditions. Values show mean ± SD (CF, *n* = 50; NF, *n* = 25). The mean ozone concentration during the daytime (6:00 to 18:00) was 2.0 nL L^{-1} in charcoal-filtered (CF) and 36.2 nL L^{-1} in non-filtered (NF) air. SL421 was a parental CSSL of SHA422-1.1 and SHA422-1.3.

			PRB	Conversion factor	Grain yield (g)	Conversion factor
2010	Sasanishiki	CF	9.4 ± 1.1	1.006	11.6 ± 2.9	1.098
		NF	10.9 ± 1.0		13.2 ± 3.3	
	Habataki	CF	11.0 ± 0.8	1.028	12.7 ± 3.5	1.001
		NF	12.9 ± 1.2		13.1 ± 3.9	
	SL421	CF	12.7 ± 1.5	0.997	12.1 ± 3.9	0.940
		NF	12.6 ± 1.0		12.5 ± 3.0	

S1 Table B. Effects of exposure to elevated ozone on grain yield and plant growth parameters in Sasanishiki and Habataki. Values show mean ± SD (n = 20). n.s., not significant; *P < 0.1; **P < 0.05 (Student's t-test). AA, ambient air; O_3, elevated ozone. Sasa, Sasanishiki; Haba, Habataki. LBS, leaf bronzing score; FGN, filled-grain number; SGN, sterile grain number; TGN, total grain number; NFP, number of filled grain per panicle; TGW, a thousand grain weight. LBS was defined using the following scale: 0, no visible damage; 1, very few small chlorotic spots; 2, very few small brown stipples; 3, 10%–20% of leaf area with chlorotic or brown stipples; 4, 20%–40% of leaf area with chlorotic or brown stipples; 5, >40% of leaf area with brown lesions; 6, >40% of leaf area with brown lesions and large necroses; 7, entire leaf dying (See [13]).

			LBS		Biomass (g)		Culm (cm)		Culm number	
2009	Sasa	AA	1.40 ± 0.55	**	62.8 ± 18.7	n.s.	90.9 ± 4.6	n.s.	8.6 ± 2.6	n.s.
		O_3	4.20 ± 0.84		61.2 ± 10.5		88.9 ± 5.6		8.5 ± 1.3	
	Haba	AA	0.00 ± 0.00	n.s.	72.9 ± 26.9	*	76.3 ± 4.8	**	3.4 ± 2.4	n.s.
		O_3	0.00 ± 0.00		65.4 ± 15.9		70.5 ± 3.3		3.5 ± 2.3	
2010	Sasa	AA	1.00 ± 1.41	**	44.6 ± 14.8	**	87.9 ± 3.2	**	6.5 ± 2.1	n.s.
		O_3	3.40 ± 0.55		56.8 ± 14.1		84.6 ± 4.5		7.2 ± 1.6	
	Haba	AA	0.20 ± 0.45	*	43.1 ± 14.9	**	73.2 ± 6.1	**	3.7 ± 0.9	**
		O_3	1.20 ± 1.10		52.7 ± 16.2		65.6 ± 4.0		4.6 ± 1.0	

			Panicle (cm)		Panicle number		FGN		SGN	
2009	Sasa	AA	18.9 ± 1.7	*	8.4 ± 2.6	n.s.	649.4 ± 194.2	n.s.	33.4 ± 22.1	**
		O_3	18.2 ± 1.1		8.4 ± 1.4		598.6 ± 113.2		52.2 ± 28.1	
	Haba	AA	23.7 ± 1.5	*	4.5 ± 1.5	n.s.	700.9 ± 262.5	n.s.	123.9 ± 56.1	**
		O_3	22.4 ± 3.1		4.7 ± 1.1		629.0 ± 154.8		205.9 ± 66.8	
2010	Sasa	AA	20.1 ± 1.0	n.s.	6.7 ± 2.1	n.s.	608.7 ± 224.1	n.s.	34.0 ± 17.3	n.s.
		O_3	20.1 ± 1.1		7.2 ± 1.6		627.1 ± 148.4		35.0 ± 18.8	
	Haba	AA	24.6 ± 1.7	**	3.7 ± 0.9	**	650.1 ± 240.0	n.s.	65.0 ± 26.0	**
		O_3	22.5 ± 1.2		4.6 ± 1.0		616.6 ± 163.3		84.3 ± 37.8	

			TGN		NFP		TGW (g)		Filling rate (%)	
2009	Sasa	AA	683.8 ± 203.1	n.s.	78.1 ± 9.0	**	21.2 ± 1.0	n.s.	95.0 ± 2.7	**
		O_3	650.8 ± 115.8		71.5 ± 8.9		21.2 ± 0.7		91.9 ± 4.5	
	Haba	AA	918.1 ± 255.4	*	154.1 ± 25.3	**	25.1 ± 1.0	**	85.1 ± 2.4	**
		O_3	788.2 ± 174.3		135.4 ± 16.5		22.5 ± 0.3		75.4 ± 5.5	
2010	Sasa	AA	642.7 ± 235.8	n.s.	92.1 ± 18.5	n.s.	23.1 ± 1.2	**	94.7 ± 0.0	n.s.
		O_3	662.1 ± 156.9		87.2 ± 10.2		22.5 ± 0.6		94.7 ± 0.0	
	Haba	AA	715.0 ± 258.6	n.s.	173.3 ± 41.7	**	23.4 ± 10.7	n.s.	90.3 ± 0.0	**
		O_3	700.9 ± 190.0		135.7 ± 22.0		19.9 ± 1.0		88.0 ± 0.0	

S1 Table C. LC conditions. MeCN, methyl cyanide (acetonitrile); SA, Salicylic acid.

Method No.	Hormone	Solvent A	Solvent B	Gradient (composition of solvent B)
1	Acid	Water containing 0.01% acetic acid	MeCN containing 0.05% acetic acid	3 to 50% 20 min
2	Base	Water containing 0.01% acetic acid	MeCN containing 0.2% acetic acid	3 to 97% 16 min
3	SA	Water containing 0.01% formic acid	MeCN containing 0.1% acetic acid	3 to 98% 10 min

S1 Table D. Parameters for LC-ESI-MS/MS analysis (Agilent 1260-6410). GA_1, gibberellin A_1; IAA, indole-3-acetic acid; ABA, abscisic acid; JA, jasmonic acid; GA_4, gibberellin A_4; JA-Ile, jasmonoyl-L-isoleucine; tZ, *trans*-zeatin; DHZ, dihydrozeatin; iP, N6-isopentenyladenine; SA, salicylic acid.

	LC method	Retention time on LC (min)	ESI	MS/MS transitions for quantifications (*m/z*)	Collision energy (V)	Fragment or (V)
GA_1<br>D_2-GA_1	1	9.0	−	347/273<br>349/275	18	160
IAA<br>D_2-IAA<br>D_7-IAA	1	10.0	+	176/130<br>178/132<br>183/135, 136, 137	10	90
ABA<br>D_6-ABA	1	12.6	−	263/153<br>269/159	05	130
JA<br>D_2-JA	1	14.4	−	209/59<br>211/59	15	135
GA_4<br>D_2-GA_4	1	17.0	−	331.2/257<br>331.2/259	18	160
JA-Ile<br>$^{13}C_6$-JA-Ile	1	18.0	−	321.2/130<br>338.4/136.2	14	140
tZ<br>D_5-tZ	2	8.5	+	220.3/136.3<br>225.3/136.3, 137.3	8	100
DHZ<br>D_3-DHZ	2	8.6	+	222.2/136.2<br>225.2/136.2	8	140
iP<br>D_6-iP	2	12.6	+	204.4/136.4<br>210.4/137.4	8	110
SA<br>D_4-SA	3	5.6	−	137/93<br>141/97	12	90

S1 Table E. Effects of exposure to elevated ozone on grain yield and plant growth parameters in SHA422-1.3, SHA422-1.1, Sasanishiki and Habataki (2011). Values show mean ± SD (n = 36). Bars topped by the same letters are not significantly different (Tukey's HSD test, P < 0.05). NF, non-filtered air (converted values); O_3, elevated ozone. Sasa, Sasanishiki; Haba, Habataki. FGN, filled-grain number; SGN, sterile grain number; TGN, total grain number; NFP, number of filled grain per panicle; TGW, a thousand grain weight.

			Biomass (g)		Culm (cm)		Culm number		Panicle number	
2011	SHA 422-1.3	NF	53.8 ± 11.7	a	86.5 ± 4.6	a	8.9 ± 1.9	ab	9.0 ± 1.9	a
		O_3	34.7 ± 10.9	b	79.4 ± 7.4	bc	7.0 ± 1.8	c	6.8 ± 2.0	b
	SHA 422-1.1	NF	45.7 ± 14.1	a	76.8 ± 5.7	cde	7.4 ± 2.3	bc	7.4 ± 2.3	b
		O_3	35.3 ± 12.3	b	74.2 ± 6.3	de	7.3 ± 2.1	c	7.1 ± 2.2	b
	Sasa	NF	49.1 ± 12.9	a	82.4 ± 4.6	ab	9.1 ± 2.6	ab	9.1 ± 2.6	a
		O_3	35.8 ± 16.3	b	78.8 ± 7.8	bcd	8.2 ± 2.9	abc	8.1 ± 3.0	ab
	Haba	NF	51.2 ± 14.3	a	72.6 ± 5.8	de	5.0 ± 1.4	d	4.7 ± 1.3	c
		O_3	32.9 ± 13.3	b	61.2 ± 8.7	f	4.8 ± 1.6	d	4.6 ± 1.6	c

			Panicle (cm)		Panicle (g)		FGN		SGN	
2011	SHA 422-1.3	NF	19.8 ± 1.0	cd	20.1 ± 4.9	a	725.2 ± 157.2	ab	102.2 ± 33.8	cd
		O_3	19.4 ± 1.6	d	12.8 ± 4.4	de	483.9 ± 176.5	de	90.6 ± 36.8	d
	SHA 422-1.1	NF	21.0 ± 1.1	b	16.8 ± 5.6	abc	607.6 ± 197.8	bcd	123.0 ± 50.8	cd
		O_3	20.6 ± 1.2	bc	14.3 ± 4.8	cde	506.9 ± 180.6	cde	191.0 ± 73.9	ab
	Sasa	NF	19.6 ± 0.9	cd	20.5 ± 5.6	a	760.4 ± 199.4	a	74.4 ± 35.3	d
		O_3	19.5 ± 1.6	cd	13.7 ± 6.7	cde	415.8 ± 278.2	e	166.3 ± 92.6	bc
	Haba	NF	24.3 ± 1.1	a	17.2 ± 5.6	ab	639.7 ± 207.0	abc	132.4 ± 53.1	bcd
		O_3	21.3 ± 3.0	b	11.8 ± 5.8	e	487.4 ± 253.8	cde	248.5 ± 197.6	a

			TGN		NFP		TGW (g)		Filling rate (%)	
2011	SHA 422-1.3	NF	817.5 ± 172.0	a	81.6 ± 12.6	cd	24.7 ± 0.7	a	88.7 ± 3.3	ab
		O_3	574.4 ± 203.8	bc	69.5 ± 10.8	d	23.4 ± 1.3	abc	83.6 ± 5.1	bc
	SHA 422-1.1	NF	721.1 ± 230.2	abc	82.8 ± 10.8	cd	23.8 ± 0.6	ab	84.0 ± 4.1	bc
		O_3	697.9 ± 244.4	abc	70.4 ± 11.3	cd	22.9 ± 1.2	abc	72.1 ± 4.8	d
	Sasa	NF	829.1 ± 220.8	a	84.4 ± 10.0	c	21.5 ± 1.0	c	91.8 ± 2.3	a
		O_3	560.7 ± 301.1	c	50.7 ± 20.7	e	22.1 ± 7.3	bc	71.9 ± 14.8	d
	Haba	NF	758.7 ± 225.0	abc	135.3 ± 26.5	a	23.1 ± 1.0	abc	82.3 ± 6.8	c
		O_3	735.9 ± 424.5	abc	101.3 ± 37.8	b	19.1 ± 3.9	d	67.2 ± 10.4	d

S1 Table F. Overrepresented attributes for the 275 genes that responded under elevated ozone condition found by gene enrichment analysis. The genes subset were extracted from microarray data by the filtering criteria as below: the fold change in Habataki ≥ 2 and that in Sasanishiki ≤ 0.5 compared with ambient air condition. The genes that have unknown functions or no GO term were not contained this data set. N_{SUBSET} and N_{ALL} indicate the number of genes in subset and microarray including GO ID, respectively. This table shows only attribute ID with $P_{\mathrm{ADJ}} < 0.05$.

Attribute ID	Attribute name	N_{SUBSET}	N_{ALL}	LOD	P value	P_{ADJ}
GO:0044699	Single-organism process	10	189	0.868	3.73E-06	0.002
GO:0009733	Response to auxin stimulus	6	48	1.224	5.19E-06	0.005
GO:0005516	Calmodulin binding	4	20	1.571	1.28E-05	0.008
GO:0007154	Cell communication	5	38	1.352	8.67E-06	0.008
GO:0007165	Signal transduction	5	38	1.352	8.67E-06	0.008
GO:0023052	Signaling	5	38	1.352	8.67E-06	0.008
GO:0044700	Single organism signaling	5	38	1.352	8.67E-06	0.008
GO:0009734	Auxin mediated signaling pathway	4	21	1.545	1.57E-05	0.011
GO:0071365	Cellular response to auxin stimulus	4	21	1.545	1.57E-05	0.011
GO:0009719	Response to endogenous stimulus	6	59	1.123	1.75E-05	0.013
GO:0009725	Response to hormone stimulus	6	59	1.123	1.75E-05	0.013
GO:0010033	Response to organic substance	6	59	1.123	1.75E-05	0.013
GO:0051716	Cellular response to stimulus	5	44	1.280	1.81E-05	0.014
GO:0009755	Hormone-mediated signaling pathway	4	23	1.498	2.30E-05	0.018
GO:0032870	Cellular response to hormone stimulus	4	23	1.498	2.30E-05	0.018
GO:0070887	Cellular response to chemical stimulus	4	23	1.498	2.30E-05	0.018
GO:0071310	Cellular response to organic substance	4	23	1.498	2.30E-05	0.018
GO:0071495	Cellular response to endogenous stimulus	4	23	1.498	2.30E-05	0.018
GO:0042221	Response to chemical stimulus	6	64	1.085	2.80E-05	0.022

S1 Table G. Effects of exposure to elevated ozone on the amounts of phytohormones (µg gFW^{-1}) in inflorescence meristems and flag leaves. Values show mean ± SD (*n* = 3). n.d., not detected. Values followed by the same letters are not significantly different (Tukey's HSD test, $P < 0.05$). IAA, indole-3-acetic acid; tZ, *trans*-zeatin; iP, N6-isopentenyladenine; SA, salicylic acid; GA_1, GA_4, gibberellins A_1 and A_4, respectively. AA, ambient air; O_3, elevated ozone.

Inflorescence meristems

		IAA		tZ		iP		SA		GA_1, GA_4
Sasanishiki	AA	47.4 ± 3.8	a	0.34 ± 0.05	a	0.18 ± 0.05	a	116.0 ± 3.3	a	n.d.
	O_3	22.4 ± 7.0	a	0.38 ± 0.09	a	0.16 ± 0.09	a	191.4 ± 11.8	a	n.d.
Habataki	AA	14.8 ± 6.9	a	0.29 ± 0.03	a	0.09 ± 0.03	a	72.0 ± 4.0	a	n.d.
	O_3	19.3 ± 0.9	a	0.47 ± 0.01	a	0.01 ± 0.01	a	182.2 ± 2.3	a	n.d.

Flag leaves

		IAA		tZ		iP		SA ($\times 10^{-3}$)		GA_1, GA_4
Sasanishiki	AA	8.35 ± 1.8	a	0.07 ± 0.03	a	0.26 ± 0.09	a	4.25 ± 1.05	a	n.d.
	O_3	1.96 ± 0.3	a	0.09 ± 0.07	a	0.47 ± 0.09	a	2.60 ± 0.32	b	n.d.
Habataki	AA	8.56 ± 1.9	a	0.08 ± 0.07	a	0.43 ± 0.39	a	2.09 ± 0.30	b	n.d.
	O_3	9.61 ± 1.6	a	0.20 ± 0.06	a	4.96 ± 0.30	b	1.77 ± 0.13	b	n.d.

S1 Fig. A. Daily ozone exposure in the glasshouse in 2009 and 2010. Values represent mean ozone concentrations, measured every hour and averaged from (○) May 21 to September 25 in 2009 and (□) June 8 to September 28 in 2010.

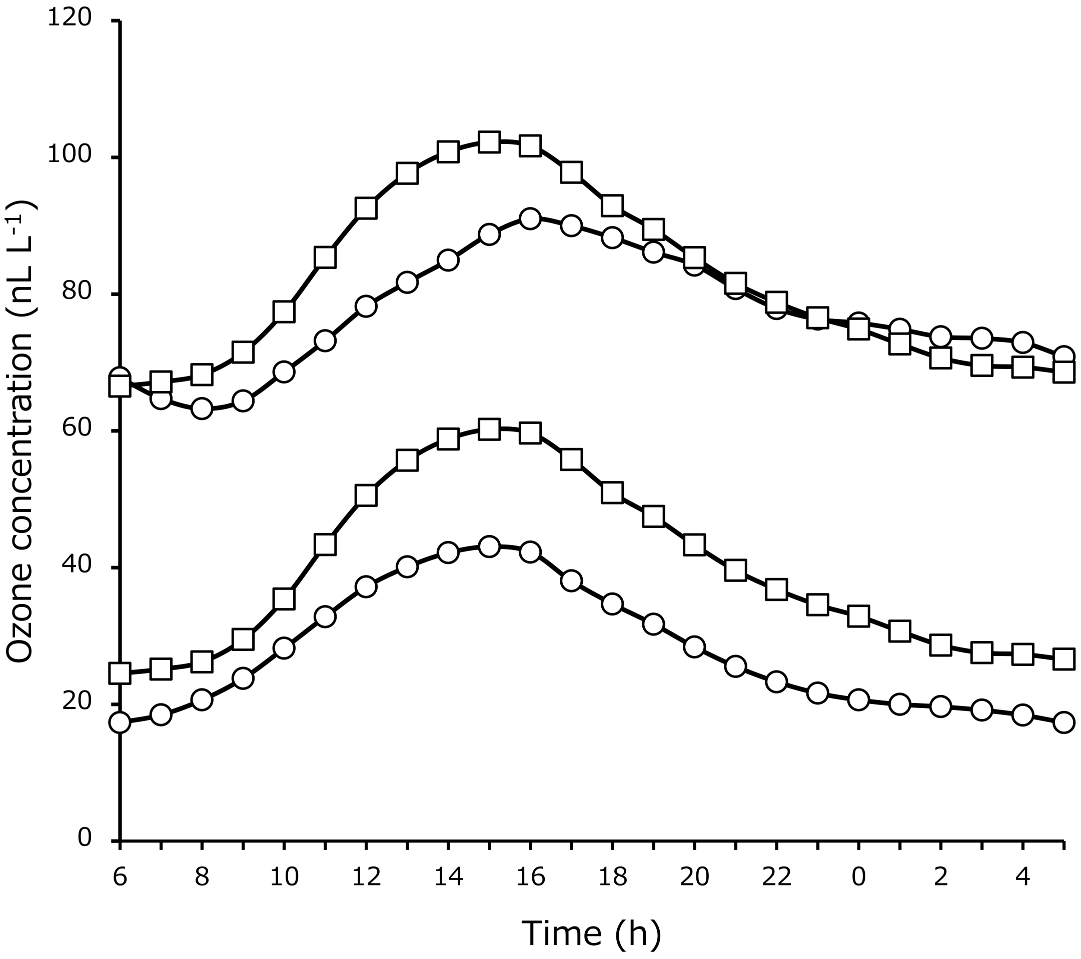

S1 Fig. B. Backbone ribbon representation of the F-box domains of APO1 from Sasanishiki and Habataki. The structures were modeled in SWISS-MODEL [25-27]. Conserved hydrophobic residues are highlighted (white). The single amino acid substitution in Habataki (Ile39Val, blue) probably does not affect the F-box domain structure.

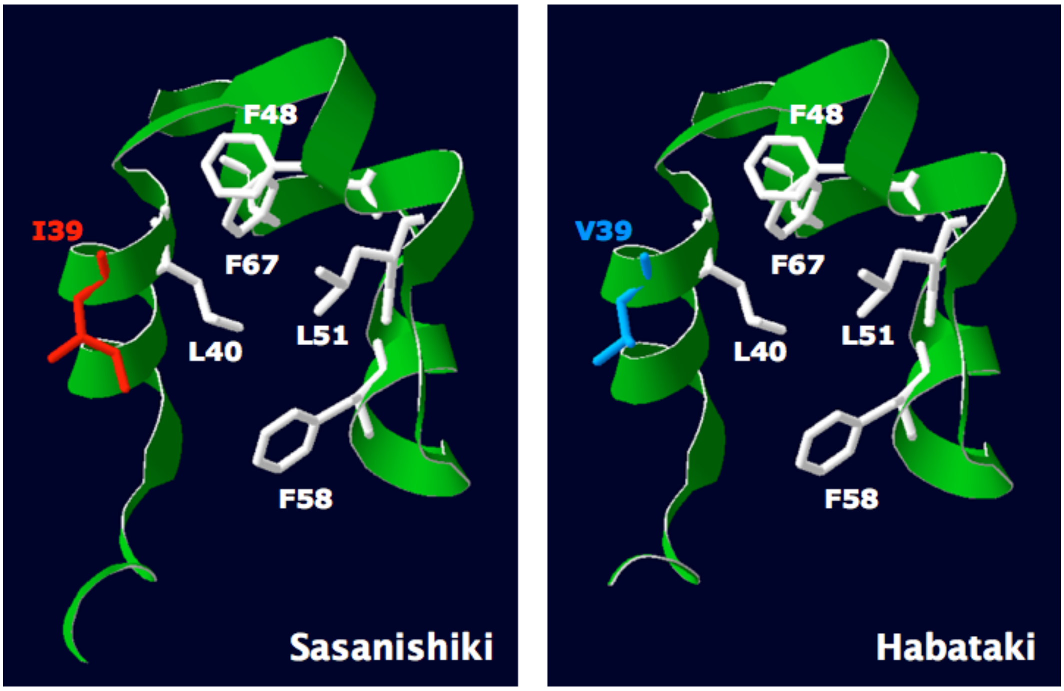

S1 Fig. C. Ozone-induced changes in the *APO1* transcript level in inflorescence meristems of SHA422-1.3 and SHA422-1.1. The data are actual measured values (*n* = 1). CF, charcoal-filtered air; O_3, elevated ozone.

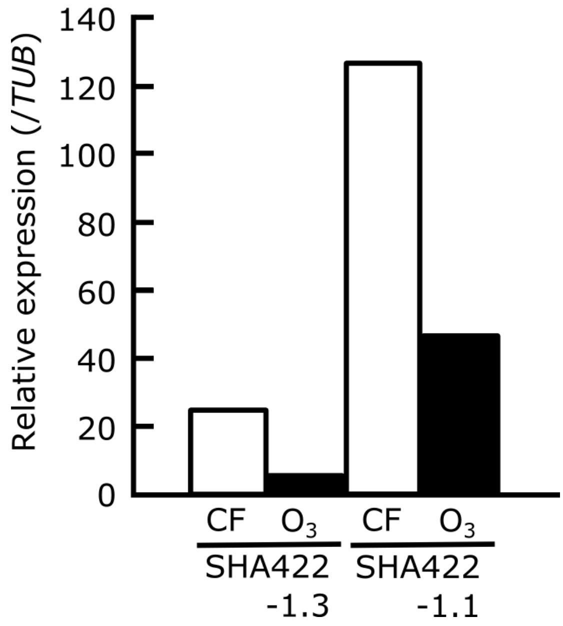

Supplement: S1 File — Table A in S1 File. Grain yield and number of primary rachis branches (PRB) grown at OTC in 2010, and conversion factors of CF to NF conditions. Table B in S1 File. Effects of exposure to elevated ozone on grain yield and plant growth parameters in Sasanishiki and Habataki. Table C in S1 File. LC conditions. Table D in S1 File. Parameters for LC-ESI-MS/MS analysis (Agilent 1260–6410). Table E in S1 File. Effects of exposure to elevated ozone on grain yield and plant growth parameters in SHA422-1.3, SHA422-1.1, Sasanishiki and Habataki (2011). Table F in S1 File. Overrepresented attributes for the 275 genes that responded under elevated ozone condition found by gene enrichment analysis. Table G in S1 File. Effects of exposure to elevated ozone on the amounts of phytohormones (μg gFW−1) in inflorescence meristems and flag leaves. Fig A. in S1 File Daily ozone exposure in the glasshouse in 2009 and 2010. Fig B. in S1 File Backbone ribbon representation of the F-box domains of APO1 from Sasanishiki and Habataki. Fig C. in S1 File Ozone-induced changes in the APO1 transcript level in inflorescence meristems of SHA422-1.3 and SHA422-1.1. (PDF) [file pone.0123308.s001.pdf]
